# Standardized Process Measures in Radiographic Hip Surveillance for Children with Cerebral Palsy

**DOI:** 10.1097/pq9.0000000000000485

**Published:** 2021-12-15

**Authors:** Kathryn S. Milks, Erin L. Mesi, Amanda T. Whitaker, Lynne Ruess

**Affiliations:** Fromt the * Department of Radiology, Nationwide Children’s Hospital, Columbus, Ohio; † Department of Orthopedic Surgery, Nationwide Children’s Hospital, Columbus, Ohio; ‡ Department of Radiology, The Ohio State University College of Medicine, Columbus, Ohio; § Department of Orthopaedic Surgery, The Ohio State University College of Medicine, Columbus, Ohio.

## Abstract

Detection of hip migration in children with cerebral palsy (CP) through radiographic surveillance can prevent dislocations. Migration Percentage (MP) is the accepted method for quantifying hip subluxation in CP on pelvis x-ray but was not being reported at our institution. Our objective was to improve care for children with CP by standardizing radiographic techniques and reporting radiographs obtained as part of a hip surveillance program.

**Methods:**

A baseline retrospective review of CP surveillance pelvis x-ray reports was performed. We then educated radiologists and technologists, standardized imaging techniques, and required structured radiology reporting to include MP measurement and dislocation risk categories. We tracked compliance with the reporting template for 10 months. Images and reports were also assessed for quality and accuracy by an orthopedic surgeon.

**Results:**

Baseline period reports showed no consistency. In total, 449 children with CP (mean age: 7.3 years ± 4.2) had a surveillance pelvis radiograph during the postintervention study period (May 2019–February 2020). An estimated 90% reporting compliance was achieved and sustained by 5 months. Eight (89%) of the children with high-risk hips were newly diagnosed during our study period; all had a progressive increase in MP from prior examinations. All clinicians surveyed agreed that the standardized reports, including MP, were helpful to their practice.

**Conclusions:**

Using evidence-based process measures and quality improvement methodology, we standardized hip surveillance for children with CP. Radiology reports that include MP and risk category for hip dislocation enable clear communication for referrals across specialties and early detection and treatment for better outcomes.

## INTRODUCTION

### Background

Children with cerebral palsy (CP) are at increased risk of hip dislocation.^1^ The incidence of hip dislocation in all patients with CP is 15%–20%, and is highest between those aged 2 and 5 years.^2,3^ Hip dislocations are a source of decreased quality of life due to pain and reduced mobility, which results in chronic degenerative change over time.^4^

Although hip migration occurs gradually over time, most patients are asymptomatic until the hip is entirely dislocated.^5,6^ Detection of hip abnormalities before dislocation requires a combination of regular hip examinations and routine radiologic evaluation.^1^ Early detection is important because once the hip has dislocated, standard surgical reconstructive options have a lower success rate due to bony and soft tissue remodeling.^5^ Early surgical intervention is also associated with better long-term hip morphology and decreased pain.^7^ Therefore, early intervention with preventative surgeries is preferred because of better long-term outcomes.^8,9^

### Local Problem

In 2017, The American Academy for Cerebral Palsy and Developmental Medicine (AACPDM) created an online evidence-based reference guide for radiographic hip surveillance based on population-based data from Sweden, Australia, and British Columbia.^9–11^ The AACPDM guidelines include recommendations for surveillance intervals, patient positioning, and a standard reporting measure of hip subluxation termed the Migration Percentage (MP). MP is recommended because it is widely validated, and literature shows patients’ hips can be risk-stratified based on MP measurement.^5^ A radiology and orthopedic collaborative was formed to determine how to implement these best practice imaging guidelines into CP hip surveillance at our institution.

Most children with CP will never be seen by an orthopedic surgeon, and care can be successfully managed by pediatricians and nonsurgical subspecialists. This, however, requires CP providers in various fields across pediatrics to screen children with CP for hip disease and know when to refer to orthopedics. Before our initiative, CP providers ordered hip surveillance x-rays, but radiologists were not performing the MP measurements, making it challenging for providers to know how to interpret the examination. Orthopedic surgeons were the only providers making MP measurements on CP hip surveillance radiographs; however, these measurements could not be saved on the image, nor were they documented in the radiology report. Standardizing radiology reports to include the MP was a pragmatic solution.

### Radiographic Technique

Before our quality improvement initiative, there were no institutional guidelines for radiology technologists regarding positioning for pelvis x-rays in CP patients. According to the AACPDM, these children should have a bolster under their knees to reduce pelvic tilt if they cannot lie flat due to hip flexion contractures. For reproducibility, patellae should be facing up, rather than placing the lower extremities in 15 degrees internal rotation, as is standard in routine non-CP surveillance pelvis radiographs.

### Reporting

MP was not part of our radiologists’ lexicon before instituting the CP surveillance structured report. Radiologists used variable terminology to describe the hips that differed from orthopedic surgeons’ descriptions, and the report “Impression” section did not include the risk of hip dislocation. Terms such as “coxa valga” and “lateral uncovering” were routinely used. In our experience, these terms are confusing to nonorthopedic physicians caring for children with CP, and their use in radiology reports often prompted unnecessary orthopedic referrals. Without specific hip migration information, referring providers did not have adequate information to determine when a patient should be referred to orthopedic surgery.

Structured reporting in radiology is used to ensure consistency and reproducibility of reporting and improve clarity.^12^ This has given rise to successful adult lesion reporting systems such as Breast Imaging-Reporting and Data System (BI-RADS) and Prostate Imaging-Reporting and Data System (PI-RADS).^13,14^ Large-scale pediatric surveillance programs, such as CP hip surveillance, also stand to gain from similarly structured reporting systems.

### Intended Improvement

Our primary goal was to improve care for children with CP by standardizing process measures in radiographic technique and reporting for pelvis radiographs obtained as part of an institution-wide hip surveillance program. The specific aim was to increase reporting of an MP for each hip on surveillance AP pelvis radiographs in children with CP to 90% and sustain for 5 months. We focused on 3 areas of intervention: (1) technologist education of appropriate patient positioning, (2) radiologist education of MP measurement, and (3) structured radiology reports.

## MATERIALS AND METHODS

### Ethical Considerations

Literature review revealed which quantitative measurements of the hip were the best predictors of subsequent hip dislocation in children with CP. The MP was agreed upon by representatives from radiology and orthopedic surgery as the single most important and practical measurement of the hip in CP because it had been validated, widely accepted, correlates with risk of dislocation, and is reproducible.^15–17^ Our institutional review board waived the need to review this quality improvement project, and the authors declare no conflicts of interest.

## SETTING

Our practice covers a large academic pediatric hospital and clinic system with more than 1.5 million patient visits, including approximately 40,000 orthopedic-related outpatient visits per year. The system employs 92 radiology technologists. In addition, radiographs are reported by 27 pediatric radiologists, regardless of subspecialty.

### Baseline Data

We queried our electronic medical record (EPIC, Verona, Wis.) monthly for all “AP pelvis” examinations performed at our institution with an indication of “CP screening,” “hip surveillance,” “cerebral palsy,” or some combination or abbreviation of these terms. Baseline radiology report data were collected over 3 months (July–September 2018) before technologist and radiologist training began in October 2018.

### Measures

**Fig. 1.**
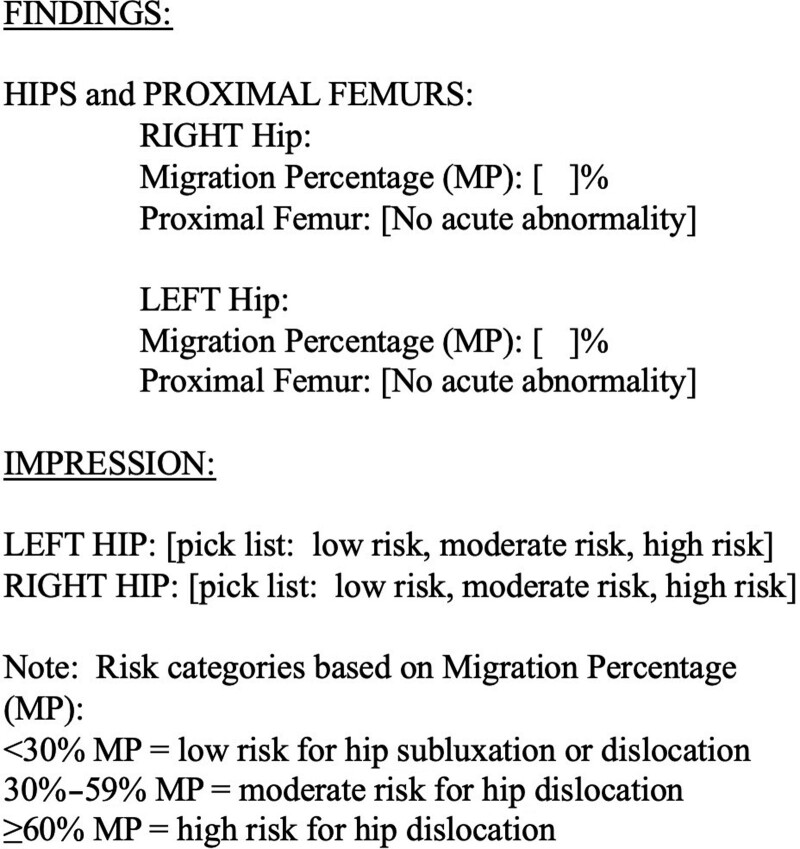
Standardized reporting template created for surveillance hip radiographs in children with CP. The Findings section requires quantification of hip migration using a numeric value, the MP. The Impression section requires a risk category assignment for each hip.

Our primary process measure was tracking compliance with the surveillance CP hip reporting template (Fig. 1). For the first 5 months postintervention, a report was considered compliant if it included the MP for each hip and did not use confusing terms (ie, “coxa valga” and “lateral uncovering”). After September 2019, a compliant report also required the risk of hip dislocation in the “Impression” section based on the MP (low, moderate, or high).

### Intervention

**Fig. 2.**
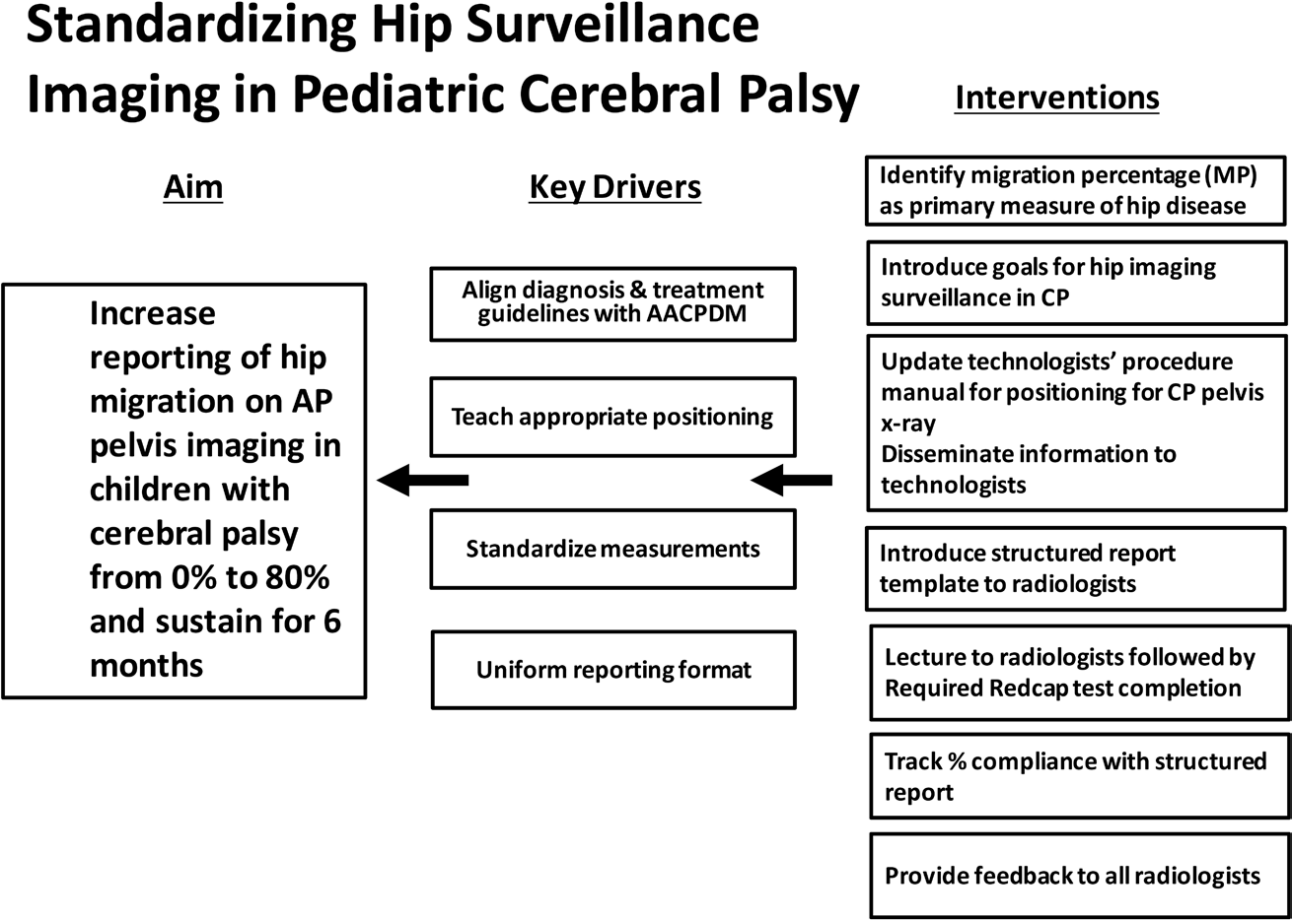
Key driver diagram for instituting and maintaining a radiographic hip surveillance program.

The QI team included lead radiology technologists, 2 board-certified pediatric radiologists, and 1 pediatric orthopedic surgeon specializing in CP hip disease. Radiologist and radiology technologist education formed the basis of our key drivers (Fig. 2).

We educated radiology technologists on patient positioning through the monthly radiology newsletter, email communication, and training meetings. In addition, our radiographic reference handbook was updated describing the technique, and positioning bolsters were made available.

**Fig. 3.**
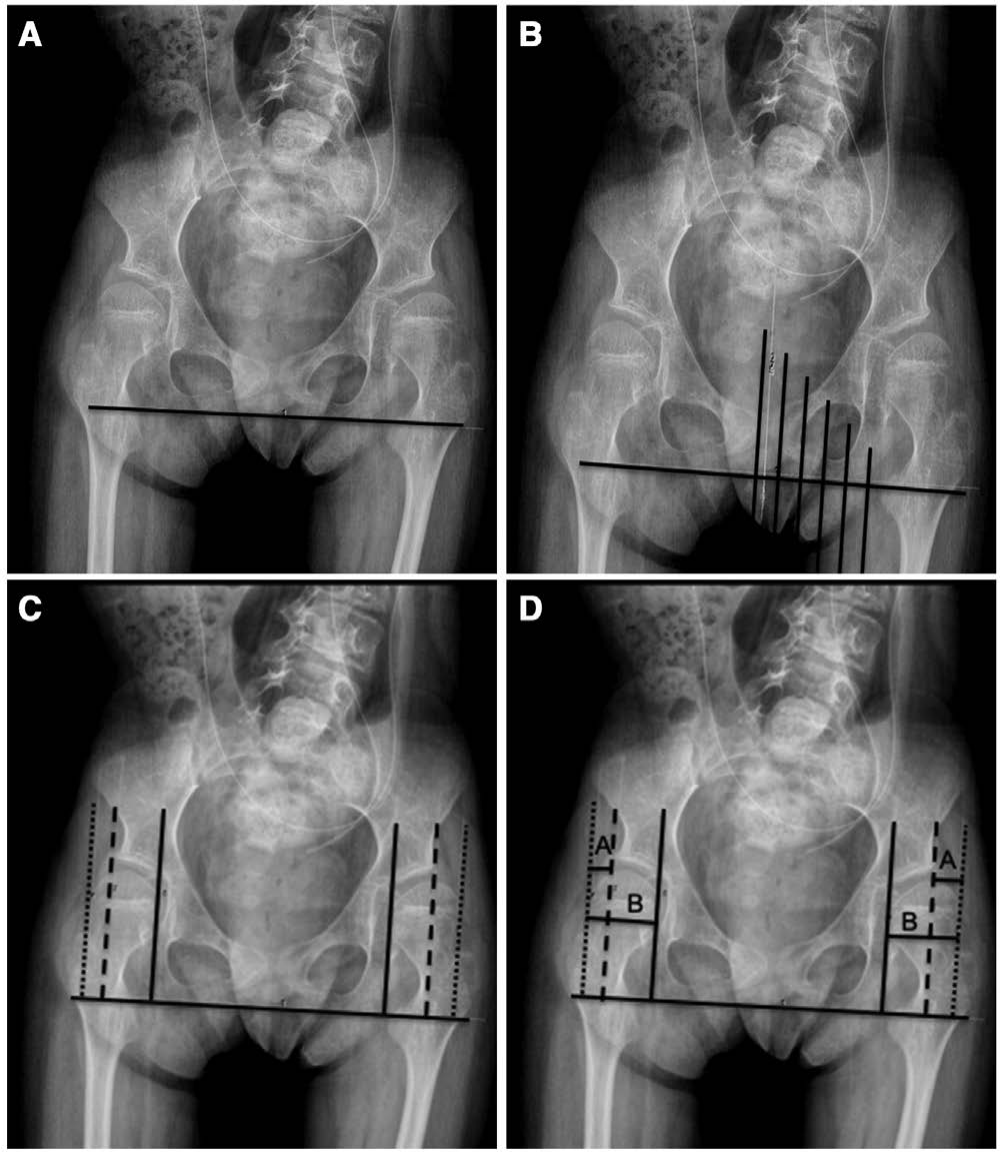
Technique for measurement of MP using the measuring tool on a picture archiving and communication workstation. a: A horizontal line along the ischia is drawn. b: Six lines are then drawn perpendicular to the ischial line. Cobb angle calculator can be used to ensure that lines are drawn 90 degrees to the ischial line. c: Two lines are moved out to frame the medial and lateral borders of each femoral epiphysis (solid line and dotted line, respectively), with the third line marinating the lateral border of the bony acetabulum (dashed line). d: Finally, horizontal lines A and B are drawn to calculate the percent femoral head lateral to the bony acetabulum, A/B × 100 = MP. *Acta Orthop Scand Suppl* 1980;184:1–1000 and *Acta Orthop* 2018;89:652–655.^18,19^

The technique for measuring the MP is shown in Figure 3. Our technique was adapted from Reimers’ original description; however, we use a line drawn horizontal to the ischial tuberosities, rather than Hilgenreiner’s line, which intersects the triradiate cartilage. This modified Reimers technique accounts for pelvic obliquity and more closely approximates hip dislocation risk.^18,19^

Radiologists received education on CP hip subluxation surveillance through didactic lectures provided jointly by radiologists and orthopedists. All radiologists were required to complete a self-directed online training module via REDCap electronic data capture tools hosted by our institution.^20^ Radiologists with noncompliant reports were given feedback and retraining tips by email communication. Additional monetary incentives for compliance with reporting templates were provided as part of our departmental bonus system.

We created a structured reporting template for surveillance CP pelvis radiographs through our reporting system (Powerscribe360, Nuance, Burlington, Mass.). During training, strict adherence to template wording was stressed to limit variability among reports.

### Data Analysis

We monitored compliance with the standardized reporting CP hip template through monthly evaluation of all AP pelvis radiographs performed in children with CP for hip surveillance. Study data were collected beginning May 2019; after all, radiologists had completed training through February 2020.

Radiology reports were reviewed and sorted manually by a pediatric radiologist (LR) with 25 years of experience using a spreadsheet (Excel, Microsoft, Redmond, Wash.) format. Patients older than 18 years (ie, those aged 18 years and 1 day) and those who had undergone corrective hip osteotomies were excluded. Examinations performed for trauma, pain, or other nonsurveillance indications were also excluded.

MP < 30% were low risk, MP 30%–59% moderate risk, and MP ≥ 60% were high risk for dislocation based on existing literature.^5,21,22^ Reports lacking MP or risk category were noncompliant because they lacked essential clinical information. Reports containing “coxa valga” or “lateral uncovering” were noncompliant because they used confusing terminology. Data before and after intervention were compared. The percentage of compliant reports was tracked over time using a run chart. Additional patient demographics and treatment by hip risk category were reviewed.

Images were reviewed for quality by an orthopedic surgeon specializing in CP hip disease (AW). Among the average 50 CP hip surveillance pelvis x-rays performed monthly, 5 (10%) were randomly selected for review. Image quality components assessed included pelvic rotation, pelvic tilt, and position of the femurs. If pelvic tilt, pelvic rotation, or femoral rotation were identified, the positioning was determined “suboptimal.” MP measurements (manual technique or using the HipScreen mobile app (App store Shriner’s Hospital for Children, Sacramento, Calif.) were compared with the radiology measurements. If the 2 measurements were within ±5 degrees of each other, they were considered as equal. We also determined whether the difference in the radiologist’s and orthopedist’s MP calculation would change the hip risk category.

In February 2021, CP providers completed a survey regarding the changes in the hip surveillance program using the REDCap electronic data capture tool hosted by our institution.^20^

## RESULTS

### Baseline Data

During the baseline period, we screened 108 children with CP with pelvis radiographs. Ten randomly sampled examinations per month showed that all children were positioned, with legs in maximal internal rotation, none with lower extremities in neutral and patellae forward, as is the accepted positioning per the AACPDM guidelines. Eighteen (17%) were reported as normal. The reports for 57 (63%) of the abnormal exams included a measurement or a numerical approximation of hip subluxation, though none reported an MP. In addition, all 90 (100%) of the abnormal exam reports included the undesired terms “coxa valga” and/or “lateral uncovering.”

### Intervention Data

#### Reporting

**Fig. 4.**
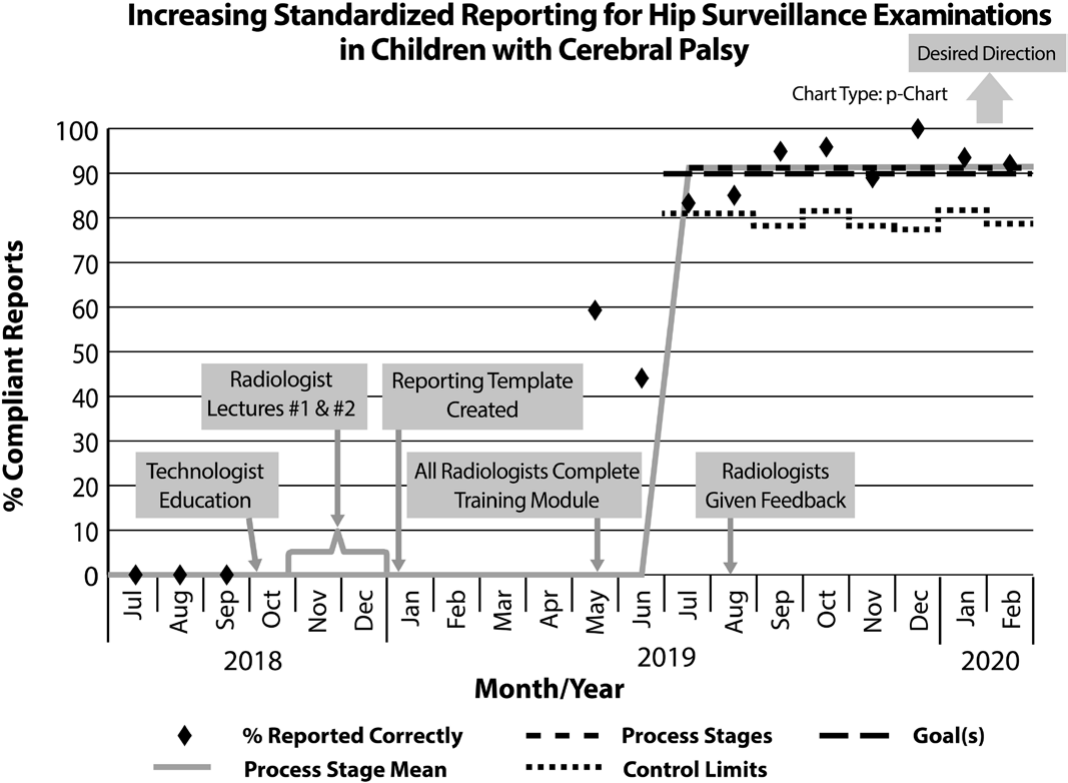
Run chart displays the variation in our process over time. By 5 months (September 2019), we had met and sustained our goal of 90% compliance with standard templated reporting in surveillance pelvis radiographs in children with CP. The dashed line indicates “goal” line of 90%. The solid line indicates the process mean. There is no shift change.

After training was complete, we tracked monthly reporting compliance for 10 months (May 2019 through February 2020) using a run chart (Fig. 4). Via electronic medical record search, we identified 526 AP pelvis radiograph reports. Twenty-six patients older than 18 years were excluded, as were 44 children who had hip surgery. Seven patients with a nonsurveillance indication for radiographs were also excluded. A total of 449 children (898 hips) were included in our study. The mean age was 7.3 years ± 4.2 (range 1–18 years, median 7 years). There were 263 (59%) men, 186 (41%) women. In the first posintervention month (May 2019), more than half (19/35, 54%) of reports were compliant. Of the noncompliant reports, 6 (38%) did not measure MP, while 10 (63%) had undesired terms. Compliance in June declined with only 41% (16/39) compliant and 59% (23/39) noncompliant. Ninety-six percent (22/23) of the noncompliant cases in June were due to use of undesired terms, with nearly 60% (13/23) of noncompliant cases read by a single radiologist. In August 2019, 86% (44/51) of reports were compliant. Among the noncompliant reports, only 2 reports (2/7, 29%) lacked the MP, and 5 reports (5/7, 71%) used undesired terms. In September 2019, the desired threshold for report compliance of at least 90% was reached [94% (34/36)].

During our 10-month study period, a total of 4 (0.8%) examinations had suboptimal radiographic technique; however, this only precluded measurement of MP in 1 child.

From September 2019 through February 2020, after hip risk categories became trackable, 275 children (550 hips) were screened. Most (507/550, 92%) hips were categorized as low (368/550, 67%) or moderate (139/550, 25%) risk for dislocation. There were 11 high-risk hips in 9 children (11/550, 2%) and 8 hip dislocations in 7 children (8/550, 1.5%) during our study period. Eight (89%) of the children with high-risk hips were newly diagnosed during our study period. All 8 showed progressive increase in MP from prior examinations. Only 1 was previously recognized as a high-risk hip. Among patients with the high-risk hip(s), corrective surgery was planned in 7 (78%) and 1 died. In the 7 children with hip dislocation (ages 2−11 years, mean age 6.3 years), 6 (86%) were newly identified. Included in this group of patients was a 11-year-old who had never been previously screened. Five hips progressed to dislocations during our study period. In this group, surgery was planned for 4 children and conservative management in 1.

#### Image Quality

By spot audit, 10 percent (5/50) of radiographs had suboptimal image quality due to positioning.

#### Measurement Accuracy

We reviewed 50 radiographs for measurement accuracy. Three radiographs lacked measurements in the report, and 2 reports included measurements for only 1 hip. Further, we reviewed 92 MP measurements. Seventy-one MP measurements were within 5% (77%), 5 of these hips bordered risk categories low/moderate (7%), while none bordered the moderate/high risk categories. Twenty-one (23%) measurements were discrepant by > 5%, with 4 (19%) MP greater than 5% difference that changed risk categories. Overall, 9 (10%) hips had conflicting risk categories (10%), with 4 hips > 5% different and most (5) < 5% different. All of these were within the low- or moderate-risk categories.

#### Survey Data

All (8/8, 100%) CP providers who routinely examine and radiographically screen hips in children with CP responded to the survey (Table 1). All (8/8, 100%) CP providers indicated that the addition of hip migration to the radiology report was helpful. Two (25%) providers stated that they ordered fewer surveillance radiographs, and 2 (25%) indicated that they referred less to orthopedic surgery based on hip radiograph reports. Four (50%) providers also made positive comments about the addition of MP to the reports.

**Table 1.** Results of the CP Providers Survey

Question	Yes (n = 8)
Do you find the addition of the hip migration percentage to the radiology report helpful?	8
If yes, how has it changed your practice? (choose 1 or more options below)	
I order more hip surveillance radiographs	2
I order less hip surveillance radiographs because I refer to orthopedics	0
I refer more to orthopedic surgery	0
I refer less to orthopedic surgery based on hip radiographs	2
Other	4 (see comments below)
(1) I always worked closely with orthopedics; so this did not change. I do feel like I have better data and better information to discuss with families.	
(2) Have better guidelines of when to refer to Orthopedics.	
(3) Greater consistency in the reports makes it easier to quickly look back to compare with prior measurements.	
(4) I have a standardized measurement tool that I can utilize between radiographers/radiology reports, along with numeric thresholds for orthopedic referrals.	

## DISCUSSION

### Radiographic Technique and Image Quality

Of the total pelvis x-rays, 10% were suboptimal per spot audit. This is less than reported by Kinch K et al in Scotland, who found 23% of AP pelvis x-rays obtained for CP hip surveillance had unacceptable positioning; however, half their studies were performed at an adult hospital with no standard CP radiographic positioning protocol. Thus, our data suggest that a CP-specific positioning protocol improves image quality.^23^ In patients with severe contractures and scoliosis, optimal positioning may be unattainable. Because each risk category encompasses a broad MP (low risk: <30%, moderate risk: 30%–59%, and high risk: ≥60%), it is unlikely that positioning alone would result in an inaccurate risk categorization. Existing literature shows that MP does not change significantly with changes in patient positioning, suggesting measurements should be made in cases with suboptimal positioning unless the necessary bony landmarks are not visible.^18,23,24^

### Measurement

Our results show that MP measurements made by radiologists and orthopedists were quite similar (75% of measurements were within 5%). This is congruent with existing CP literature. Parrott et al quantified a standard measurement error for MP of 6%, and Shore et al quantified the mean absolute difference in MP measurements as <7%.^25,26^ In our population, even those MP measurements that differed, infrequently resulted in a change in the hip risk category, and if they did, it was predominantly in the low- to moderate-risk group. Since a low- or moderate-risk hip is less likely to need surgery, a discrepant measurement may not be clinically significant in this group.

### Reporting

To our knowledge, we are the first institution to standardize hip surveillance imaging reports and include risk categories. Willoughby et al identified inconsistent radiology reporting as the single most frequently cited barrier to performing hip surveillance by CP providers (35%).^27^ Furthermore, even if radiologists routinely report the MP, the ordering clinician must still know how to interpret that number, and how to act on any given value. This prompted us to include a dislocation risk category for each hip based on the MP. Through our initiative in standardized reporting, clinicians can categorize the degree of hip migration, track change rate over time, and quickly identify patients at the highest risk who need a prompt orthopedic referral to prevent a hip dislocation from occurring. Per the AACPDM guidelines, any child with a hip MP > 30% should be referred to orthopedics.^10^ Our data showed that although high-risk hips are relatively rare (<5% of our surveillance population), nearly all (90%) were newly detected as part of our surveillance program, suggesting they would have otherwise been missed if the program were not in place.

### CP Provider Survey

When surveyed 2 years after implementing standard reporting, all CP providers indicated the change was helpful in their clinical practice. Their comments suggested a better understanding of screening and referral guidelines with our interventions. They also noted greater consistency in the reports allowed for accurate comparison over time and provided data for patient-family discussions. Provider feedback reinforces that uniform and succinct radiographic reporting language positively impacts patient care by guiding CP providers down the appropriate clinical care pathway, either continued radiographic surveillance or orthopedic referral.

#### Limitations

One limitation of our study was that we chose to exclude patients who had undergone corrective hip surgery. When we began, we were unaware that hip surveillance is still recommended postoperatively, and thus did not include these patients in our study. We have since learned that surgery decreases, but does not eliminate, the risk of hip dislocation in high-risk groups.^9^

## CONCLUSIONS

Using evidence-based process measures and quality improvement methodology, we standardized hip surveillance imaging and reporting for children with CP at our institution. Our standardized reporting in CP hip surveillance has resulted in early and accurate detection of children at risk for hip dislocation. In addition, radiology reports that include MP and risk category for hip dislocations enable clear communication for referrals across specialties, and earlier detection facilitates prompt treatment for better outcomes.

## DISCLOSURE

The authors have no financial interest to declare in relation to the content of this article.
